# Implementation of an interprofessional medication adherence program for HIV patients: description of the process using the framework for the implementation of services in pharmacy

**DOI:** 10.1186/s12913-018-3509-8

**Published:** 2018-09-10

**Authors:** Mélanie Lelubre, Olivier Clerc, Marielle Grosjean, Karim Amighi, Carine De Vriese, Olivier Bugnon, Marie-Paule Schneider

**Affiliations:** 1 Community Pharmacy, School of Pharmaceutical Sciences, University of Geneva, University of Lausanne, Geneva, Switzerland; 2 Community Pharmacy, Department of Ambulatory Care and Community Medicine, University of Lausanne, Lausanne, Switzerland; 3 Department of Pharmacotherapy and Pharmaceutics, Faculté de Pharmacie, Université libre de Bruxelles (ULB), Brussels, Belgium; 4 Department of Internal Medicine and Infectious Diseases, Pourtalès Hospital, Neuchâtel, Switzerland

**Keywords:** Implementation process, Implementation strategies, FISpH, Medication adherence, IMAP, Community pharmacy service, Interprofessionality, HIV patients

## Abstract

**Background:**

The community pharmacy center of the Department of Ambulatory Care and Community Medicine of the Policlinique Médicale Universitaire (PMU), Lausanne, Switzerland developed and implemented an interprofessional medication adherence program for chronic patients (IMAP). In 2014, a project was launched to implement the IMAP for HIV patients in a public non-academic hospital with the collaboration of community pharmacists in the Neuchâtel area (Switzerland). This article aims to describe the different implementation stages and strategies of the project.

**Methods:**

A posteriori description of the implementation process, including the conceptualization strategies and stages (exploration, preparation, operation, sustainability) using the Framework for the Implementation of Services in Pharmacy (FISpH).

**Results:**

In 2014, an attending infectious disease physician and a nurse at a public hospital (Neuchâtel, Switzerland) contacted the PMU to implement the IMAP in their setting in collaboration with community pharmacies. Five volunteer community pharmacies in Neuchâtel were trained to deliver the program. Three factors were found to be essential to the successful launch and progress of the implementation project: the experience of the community pharmacy center of the PMU with the IMAP, the involvement of the PMU research team, and collaboration with an external start up (SISPha) to train and support pharmacists. During the operation stage, the most important strategy developed was that of regular meetings between all stakeholders. These allowed healthcare professionals to discuss the implementation progress, to address each stakeholder’s expectations, and to exchange experiences to facilitate interprofessional collaboration and program delivery. Structural changes allowed the formalization of the activities at the hospital and in a community pharmacy. This formalization was identified as the transition step between the operation and the sustainability stages.

**Conclusions:**

The transfer of the IMAP for HIV patients to a non-academic setting and its implementation are feasible. However, implementation of a new model of pharmacy service such as IMAP implies a deep change in practice. A transitional external support and the allocation of sufficient resources to carry out the IMAP are essential for its long-term sustainability.

## Background

An innovative interprofessional medication adherence program (IMAP) was developed and implemented in the community pharmacy center of the Department of Ambulatory Care and Community Medicine at the Policlinique Médicale Universitaire (PMU), Lausanne, Switzerland [1]. In 2004, the program was developed in collaboration with the infectious diseases service of the University hospital (CHUV, Lausanne, Switzerland) and focused on HIV-positive patients. In Switzerland, around 20′000 persons live with HIV [2]. In the context of a transmitted disease with potential development of treatment resistances, support of adherence to antiretroviral treatments takes on its full meaning. Initially, this program was confined to the community pharmacy of the PMU. In 2008, its research team attempted to implement its medication adherence program for hypertensive, diabetic and/or dyslipidemic patients in community pharmacies in the French-speaking part of Switzerland [3]. The study failed and detected four major barriers to implementation: a) lack of pharmacist-patient communication, which led to insufficient promotion of the program; b) insufficient collaboration between pharmacists and physicians; c) difficulties in integrating the program into the routine activity of the pharmacy; and d) lack of motivation among pharmacists. In 2014, at the request of an infectious disease physician and a nurse, a project was launched to implement the IMAP at the public hospital ‘Hôpital neuchâtelois Pourtalès’, in collaboration with community pharmacists in the Neuchâtel area (Switzerland). Compared to the CHUV, Pourtalès hospital is a non-academic state hospital with around 190 beds that serves the entire county (178,434 inhabitants in 2016). The hospital infectious diseases outpatient ward manages about 200 HIV-infected patients [4].

Implementation research in the field of pharmaceutical care has become essential to ensure transfer of pharmacy services in the real world, program effectiveness and fidelity of the service delivery. Several implementation frameworks exist in the literature, for example the Re-AIM (Glasgow [5]), the Consolidated Framework for Implementation Research (Damschroder [6]), the Proctor Conceptual Model for Implementation Research (Proctor et al. [7]), and the Exploration, Preparation, Implementation, Sustainment Model (Aarons et al. [8]). They are, however, not specific to pharmaceutical care interventions.

In 2015, a new implementation framework specifically designed for community pharmacists emerged in the literature. This was the Framework for the Implementation of Services in Pharmacies (FISpH) developed by Moullin et al. [9]. The FISpH was based on a literature review of existing frameworks, from which a qualitative study was conducted to contextualize and advance the concept framework developed for pharmacy services [10, 11]. The framework states that implementation is a complex multi-stage process involving an innovation, a multi-level context (individuals, pharmacy(s), local setting and system), influenced by a range of factors, strategies, and evaluations [9].

In Switzerland and worldwide, implementation studies are scarce and new pharmaceutical services encounter difficulties to be implemented in community pharmacies, hence inhibiting their uptake [10, 12]. Implementation science is developing and there is a need for data in the field of community pharmacy services, particularly concerning implementation processes and used strategies [12]. Therefore, this article aims to describe, the different stages and the implementation strategies developed to implement the IMAP in a new clinical setting using the FISpH.

## Method

### Study design

This is a descriptive, observational study, focusing on the a posteriori description of the implementation process to conceptualize strategies and stages used during the study, using the FISpH framework [10]. In this project, the research team was involved in both the development of strategies to support the implementation process and in the prospective observation of the implementation results. Quantitative and qualitative results of the prospective observational study for this implementation project will be described separately in another article.

### Framework used for describing the implementation process

The FISpH framework was chosen because its development emerged from a thorough review of the existing literature was adapted in a unique way for community pharmacy services [10]. It divides the implementation process into five stages: exploration, preparation, testing, operation, and sustainability. These stages are preceded by development of the innovation and by communication about it.

### Described stages

In this paper, the context and strategies have been investigated in relation to four FISpH stages out of the five: 1) exploration (whereby the end users appraise the innovation and decide to either accept/adopt or reject it); 2) preparation (the course of preparation prior to innovation use), 3) operation (innovative use of services and their process of being integrated into routine practice through active and planned approaches), and 4) sustainability (process of maintaining the innovation that has been integrated as routine practice through continued innovation, ongoing capacity and supportive environment, and persistence of innovation benefits) [10]. The testing phase, which in this project could be considered as the inclusion of the first patients, is not described because no real transition from the testing to the operation phase was planned.

The dissemination of the program, defined as the “active approach using planned strategies via determined channels to persuade the target audience to adopt new innovations” [10], is part of the communication. This is described in both the preparation stage and the last stage of this project. Hence, communication strategies allow each new professional who wishes to adopt the intervention to enter a new implementation cycle at the exploration stage, and then to implement it at his or her individual level.

### Description of the implemented intervention

The IMAP medication adherence program was initially developed at the community pharmacy center of the PMU. In 2004, the program became a routine pharmacy intervention when the collaboration with the infectious diseases service of the Lausanne University Hospital (CHUV) was launched. The program aims to support and to reinforce medication adherence through multifactorial and interprofessional intervention. Motivational interviewing is combined with medication adherence electronic monitoring and feedback to the patient (MEMS™ monitors, Aardex MWV, Switzerland), while the core content of the pharmacist’s intervention is reported to the patient’s physician and nurse. Patient-pharmacist interviews last approximately 15 min. They are repeated over time according to the patient’s needs [1]. The intervention is based on socio-cognitive theory, especially the information-motivation-behavior skills model [13]. The program and its development have been fully described elsewhere [1].

SISPha, a Swiss startup active in the development of interprofessional programs dedicated to chronic care management, scaled up the IMAP. SISPha trains community pharmacists and pharmacy teams. It developed an interprofessional web platform for collecting patients’ data related to medication adherence [14]. In the French-speaking part of Switzerland, all pharmacies can subscribe to the SISPHA program, be trained in it and receive a toolbox to support their patients’ medication adherence. In Switzerland, community pharmacist activity to support medication adherence in chronic patients who are prescribed at least three concomitant chronic drugs are reimbursed by a fee defined by the Swiss healthcare system [1].

## Results

**Fig. 1:**
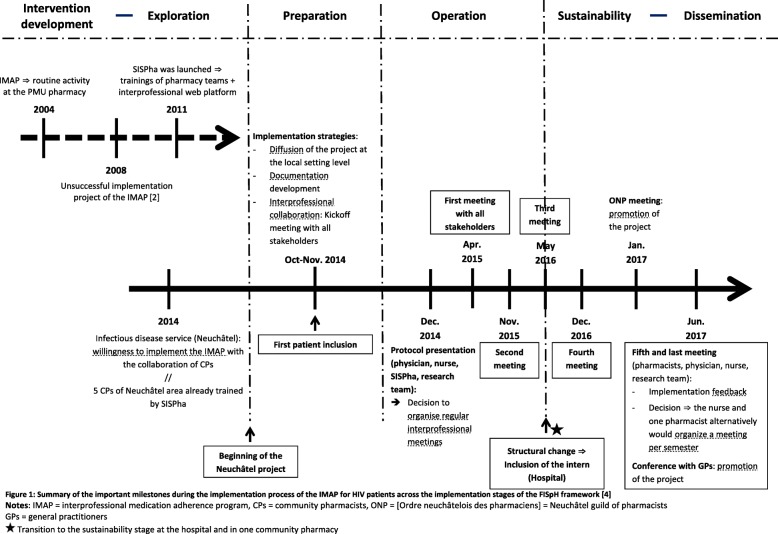
Summary of the important milestones during the implementation process of the IMAP for HIV patients across the implementation stages of the FISpH framework [12]. This figure describes all important events and strategies developed to implement the IMAP at the hospital and in community pharmacies. Events are enumerated on a timeline and separated by dotted lines on the basis of the implementation stages

Figure 1 summarizes the timeline and important milestones that drove the project forward across the implementation stages.

### Exploration

In 2014, the attending infectious disease physician (named physician in the manuscript) and a nurse working together in the district hospital Hôpital neuchâtelois, Pourtalès (Neuchâtel, Switzerland) contacted the PMU community pharmacy center. Both professionals were confronted with non-adherent patients in their practice and expressed their willingness to implement the IMAP in their hospital with the collaboration of community pharmacists in Neuchâtel (Switzerland).

When the project was launched, five community pharmacies in Neuchâtel had already decided to adopt the practice innovation supported by SISPha and were ready to deliver the program. Unlike the PMU community pharmacy center, these pharmacies are not affiliated to a research or academic structure.

### Preparation

Key stakeholders were identified and engaged in the implementation of the project from its early stage. These were a physician, a nurse, five pharmacists and SISPha collaborators.

SISPha training courses are described in Table 1. They are not mandatory to deliver the program. However, all five pharmacists had at least followed the two-day initial course “Medication adherence program: first steps at the pharmacy – parts 1 & 2” and received the in-situ training at the pharmacy before or during the project. One pharmacist attended all available courses.

**Table 1:** Description of SISPha training courses and pharmacist participation during the preparation stage

Title	Course objective	Who can attend the course?	Course duration	Number of trained pharmacies^a^
Medication adherence program: first steps at the pharmacy (part 1)	Understanding medication adherence and related issues – defining and developing strategies to implement the program in the pharmacy setting	Pharmacists	1 day	5/5
Medication adherence program: first steps at the pharmacy (part 2)	Building up competencies in presenting the program to patients and physicians	Pharmacists	1 day	5/5
The medication adherence interview	Initiation in motivational interviewing; learning about available tools for monitoring and supporting medication adherence	Pharmacists	2 days	3/5
Proposal and follow-up interviews	Building up competencies in engaging patients in the program and in conducting medication adherence interviews	Pharmacists	1 day	1/5
What is the role of the pharmacy technician?	Training in uploading and managing EM^b^ data on SISPha web platform	Pharmacy technicians	4 h	0/5
A team story	In-situ entire team training at the pharmacy	Pharmacy team	2 h30	5/5

^a^Among the pharmacies involved in the project (*n* = 5)

^b^*EM* electronic monitor

Before launching the program, different preparatory strategies were developed to inform all community pharmacists in the Neuchâtel area and physicians at the hospital about the project and to prepare the necessary documentation (Table 2 and Fig. 1). To start the operation stage officially and to develop an interprofessional collaboration, a kickoff meeting between the physician, the nurse, the five pharmacists, SISPha and the research team was organized.

**Table 2:** Description of the strategies developed during the preparation stage of the IMAP implementation

Interventions	Provision level	Developed strategies
Dissemination of the project	Local setting (pharmacies)	Identification of trained community pharmacists and encouragement of new pharmacists to receive training in medication adherence (e.g. SISPha program)
	Local setting (hospital)	Presentation of the medication adherence program to inform all healthcare professionals (physicians and nurses) at the local hospital
Development of the documentation by the research team	Organization (hospital)	“Descriptive document of the program” for the physician and the nurse to help them present the program to patients, including the list of trained pharmacists
	Organization (hospital)	A check-list for the physician and the nurse for collecting patient inclusion data. Collected data were: 1) proposal date; 2) acceptation or refusal of inclusion; 3) reason for proposal; 4) reason for refusal or chosen pharmacy for the program, whichever applies
	Organization (research)	The study protocol elaborated by the research team to evaluate the implementation outcomes; this was reviewed and approved by the physician, the nurse and SISPha
Development of the interprofessional collaboration	Organization (pharmacists, physician, nurse, SISPha, research team)	Organization of a kickoff meeting between all stakeholders to discuss and align the expectations of all stakeholders, and to present the study protocol

### Operation

The IMAP started in Neuchâtel in November 2014 (Fig. 1). The roles of each stakeholder are described in Table 3.

**Table 3:** Respective roles of each involved stakeholder during the operation stage of the IMAP implementation process

Stakeholders	Roles
The physician and the nurse	Include patients in the program and refer them to a pharmacist, who delivers the IMAP
Pharmacists	Deliver the IMAP and send the medication adherence report to the physician and the nurse
SISPha	Train pharmacists and provide the necessary tools (web platform and electronic monitors)
The research team	Organize regular meetings with stakeholders, support the implementation process and establish the collaboration between the pharmacists and the physician-nurse dyad

During this stage, it was decided to organize meetings every six months between all involved stakeholders. These meetings were held to discuss the implementation progress and each stakeholder’s expectations, and to exchange experiences to facilitate the interprofessional collaboration and the program delivery. As the PMU research team was also involved in these sessions, it was an opportunity to exchange experiences and share the IMAP guidelines as implemented at the PMU. The content of these meetings was adapted according to the implementation progress and the healthcare professionals’ feedback and needs. Each issue detected during the discussion was addressed and then reviewed during the next meeting. The research team also supported the physician and the nurse in the patient inclusion process through a monthly teleconference with the nurse to evaluate the inclusion process and related issues.

The nurse spontaneously took the lead at the hospital. She identified eligible patients with the physician and was in charge of the administrative work related to the project. A structural organizational change in the hospital lengthened the stay of the ward’s visiting intern, an infectious disease fellow, to six months. This allowed the physician and the nurse to include the intern in the program. To do so, the nurse developed local procedures and started to train the new intern. Analysis of the project by the research team found that this improvement was the pivotal event in the transition between the operation and the sustainability stages. Among the pharmacists, only one pharmacist reorganized internal activity, which she did upon the arrival of a new pharmacist during the project. This team organization allowed the participating pharmacist to enter a routinization phase and then to move to the sustainability stage.

### Sustainability

During the sustainability phase, the nurse and the physician extended the program to hepatitis C patients. A fourth meeting with all stakeholders was organized two years after the first patient was included to discuss the sustainability of the program. To reinforce this sustainability, the healthcare professional team decided to hold an interprofessional meeting at the hospital each semester, to be organized alternately by the nurse and one pharmacist. Here, the pharmacists would present one or two complex clinical cases. These cases would be discussed with the physician and the nurse to agree on an interprofessional follow-up of the patient. The first interprofessional meeting – without the research team – took place in November 2017. Two physicians, one nurse and four pharmacists took part.

### Dissemination

The program and the first results of the implementation study were presented to the board of the local pharmacists’ association (Ordre Neuchâtelois des Pharmaciens – ONP) two years after the first patient was included. This meeting was organized to promote the project and encourage more pharmacists to provide the IMAP in their practice. A letter summarizing the implementation results was sent to all pharmacists in the Neuchâtel area through the ONP. At the hospital, the research team presented the results of this project to the general practitioners in the Neuchâtel area at a conference in order to enhance the program’s adoption.

## Discussion

This project aimed to implement the IMAP in the infectious diseases department of the local hospital of Neuchâtel area with the collaboration of community pharmacists. The experience of the PMU community pharmacy with the IMAP program, the involvement of the PMU research team – who supported and evaluated the implementation process – and the collaboration with SISPha – who trained and supported the pharmacists – were essential to launch and push the implementation project forward. Two important strategies allowed stakeholders to move the project forward: 1) regular meetings between stakeholders to develop interprofessional collaboration and teamwork; and 2) the formalization of the activity at the hospital and in one pharmacy through the involvement of new colleagues, made possible thanks to structural changes (e.g. the infectious disease fellow’s six-month internship). Importantly, our results also show that such a process takes time as two years were needed to reach structural changes.

In the literature, different strategies are described to facilitate the implementation of new services. Powell et al. published a compilation of 75 implementation strategies [15]. Some of these were used during the preparation stage of our IMAP implementation: 1) develop an academic partnership; 2) assess for readiness and identify barriers and facilitators; 3) conduct education meetings; and 4) develop and organize quality monitoring. Other strategies were used during the operation and sustainability stages of the IMAP implementation: 1) build a coalition (recruit and cultivate relationships with partners in the implementation effort); 2) provide feedback in implementation team meetings; and 3) remind clinicians to deliver the medication adherence program. According to the Watkins et al. study, the most effective strategies for implementing clinical guidelines in community pharmacies are educational interventions and computerized decision support [16]. In this project, the courses and the web software developed by SISPha helped pharmacists implement the program in their pharmacies. However, based on our observations, the most useful strategy to develop interprofessional collaboration, exchange experiences and align expectations, and give feedback on the implementation progress was the organization of regular meetings with all stakeholders. These meetings helped to create a positive atmosphere and nurture the climate for change. The implementation climate is defined by Klein and Sorra as “the extent to which intended users perceive that innovation use is expected, supported, and rewarded” [17]. In our program, the future challenge for the involved healthcare professionals is to maintain the established dynamic.

Sustainability is the least described stage in the literature and many projects have failed to continue to deliver a new intervention on the long term [18]. In Neuchâtel, the sustainability stage was reached, and the project continued to be delivered after the end of the study. The two-year support provided by the research team was essential for this project and generated information about the program’s sustainability. This support is not feasible in the long run and was therefore transferred to the healthcare professionals. Long-term monitoring, without research support, has been put in place by the research team to assess the capacity of healthcare professionals to maintain the program in their practice.

This project had several limits. First, the project started exclusively with innovators as it included only one physician, one intern, one nurse and five pharmacists. It will be important to monitor the uptake of the project by other pharmacists, physicians and nurses. Another limit was that the transition between the testing and the operation stages was not clearly defined and thus not described in this paper. A clear definition and the evaluation of a testing stage could bring additional information and could improve the implementation progress by overcoming barriers detected during this stage. However, we used the results of an unsuccessful implementation study that we carried out in the past as a testing phase [3]. The four barriers that emerged from this former experience were removed by the active collaboration of all stakeholders and the proposed medication adherence training for pharmacists. Moreover, the organization of regular meetings allowed the research team to assess and overcome barriers in a timely and efficient way. In the future, the feasibility of these regular meetings should be investigated on a larger scale. The dissemination of the IMAP to a larger scale started with the communication of the information to other healthcare professionals and by addressing another pathology than solely HIV (i.e. hepatitis C). A larger study is currently being supported by the national government in Switzerland to implement an integrated chronic care management model combining the IMAP with the treatment plan reconciliation in patients with diabetes type 2 [19]. This study should bring more results on IMAP implementation in daily routine. Pharmacists involved in the HIV IMAP implementation project also started this new study in diabetes.

## Conclusion

The transfer of the IMAP for HIV patients to a non-academic setting and its implementation are feasible. The implementation of a new model of pharmacy service such as IMAP implies a deep change in practice, e.g. organizational changes, development of communication between healthcare professionals, and changes in mentalities. Moreover, the medium-term external support of an implementation research team and the allocation of sufficient resources are essential for the long-term sustainability of such a project.

## Abbreviations

CHUV: Centre Hospitalier Universitaire Vaudois (Lausanne University Hospital), Lausanne, Switzerland [20]
FISpH: Framework for the Implementation of Services in Pharmacy
IMAP: Interprofessional medication adherence program
ONP: Ordre Neuchâtelois des Pharmaciens, pharmacist association of Neuchatel county (178,434 inhabitants at the end of 2016) [21]
PMU: Policlinique Médicale Universitaire); Department of Ambulatory Care and Community Medicine (Lausanne, Switzerland) [22]
